# Supplementary material for: BMP Signaling Interferes with Optic Chiasm Formation and Retinal Ganglion Cell Pathfinding in Zebrafish
Source: Int J Mol Sci. 2021 Apr 27;22(9):4560. doi: 10.3390/ijms22094560 (PMC8123821; doi:10.3390/ijms22094560)
Supplement: Supplementary file 1 [file ijms-22-04560-s001.zip › ijms-1186269-supplement.pdf]

Supplementary Table S1: Results of a FIMO search for transcription factor binding motifs in the putative promotors of selected genes. Motifs Mad and SMAD5 are presumptive BMP-responsive elements; GLI2 is a presumptive Shh-responsive element.

Motif ID	Alt ID	Sequence Name	Strand	Start	End	p-value	q-value	Matched Sequence
SMAD5	MA1557.1	shha	+	1994	2003	4.95E-05	1	CGTCGAGCCA
GLI2	MA0734.1	shha	-	384	395	0.000149	1	AAGCCCACACGG
SMAD5	MA1557.1	shha	+	2248	2257	0.000153	1	CGCCTCGACA
Mad	MA0535.1	shha	+	2329	2343	0.00024	1	TTGACGAGAGTGCTG
SMAD5	MA1557.1	shha	-	1994	2003	0.00025	1	TGGCTCGACG
Mad	MA0535.1	shha	+	2281	2295	0.000297	1	CGCTCGAGAACACCG
SMAD5	MA1557.1	shha	+	828	837	0.00032	1	AGTCTAGAAA
Mad	MA0535.1	shha	-	1988	2002	0.000414	1	GGCTCGACGGGACGA
GLI2	MA0734.1	shha	-	676	687	0.00045	1	TGGACCACGGAA
Mad	MA0535.1	shha	+	2249	2263	0.000466	1	GCCTCGACAACGAGA
SMAD5	MA1557.1	shha	-	2375	2384	0.00048	1	AGTCCGGACA
SMAD5	MA1557.1	shha	+	2375	2384	0.000493	1	TGTCCGGACT
SMAD5	MA1557.1	shhb	+	1923	1932	8.54E-05	1	CGTCGAGTCA
Mad	MA0535.1	shhb	-	804	818	0.000112	1	GGGGCGCGAAGGAGC
GLI2	MA0734.1	shhb	-	638	649	0.000221	1	GGGACCACTTGG
SMAD5	MA1557.1	shhb	-	88	97	0.00025	1	TGGCCAGACT
Mad	MA0535.1	shhb	+	2153	2167	0.000272	1	CGCGCGAGAAGACCC
SMAD5	MA1557.1	shhb	+	88	97	0.000302	1	AGTCTGGCCA
SMAD5	MA1557.1	shhb	+	528	537	0.000335	1	AGTCTAAACA
SMAD5	MA1557.1	shhb	-	1923	1932	0.000348	1	TGACTCGACG
GLI2	MA0734.1	shhb	-	2121	2132	0.000352	1	ACGACCACTACC
Mad	MA0535.1	shhb	-	1917	1931	0.000369	1	GACTCGACGGAGAGC
Mad	MA0535.1	shhb	+	2151	2165	0.000457	1	TTCGCGCGAGAAGAC
SMAD5	MA1557.1	ephb2b-201	-	2078	2087	6.23E-05	1	AGTCCAGACA
SMAD5	MA1557.1	ephb2b-201	+	2078	2087	8.46E-05	1	TGTCTGGACT
Mad	MA0535.1	ephb2b-201	-	1924	1938	0.000179	1	CAGACGAGGGGAGGA
SMAD5	MA1557.1	ephb2b-201	+	578	587	0.000195	1	GGTCCAGACT
SMAD5	MA1557.1	ephb2b-201	-	578	587	0.000235	1	AGTCTGGACC
Mad	MA0535.1	ephb2b-201	-	2029	2043	0.000284	1	CAGACGCTGGCGCAG
GLI2	MA0734.1	ephb2b-202	+	847	858	0.000394	1	TGGCCCACCATA

SMAD5	MA1557.1	ephb2b-202	-	36	45	0.000444	1	AGACTAGCCT
SMAD5	MA1557.1	ephb2b-202	+	36	45	0.000485	1	AGGCTAGTCT
Mad	MA0535.1	lrig1-201	-	1509	1523	4.49E-05	0.722	CTCACGCCAGCACGG
Mad	MA0535.1	lrig1-205	-	524	538	4.49E-05	0.722	CTCACGCCAGCACGG
Mad	MA0535.1	lrig1-205	+	2004	2018	0.000141	1	GCGGCGTGGCTGGGA
SMAD5	MA1557.1	lrig1-205	+	2207	2216	0.000455	1	TGTCCAGTCC
Mad	MA0535.1	rgmd	-	1229	1243	2.73E-05	0.722	CCCACGCGAACGCAG
Mad	MA0535.1	rgmd	-	1445	1459	0.000408	1	CAGCCGCCGCAGCGG
Mad	MA0535.1	sema3d	+	1986	2000	2.66E-05	0.722	GACACGACGGCACAG
Mad	MA0535.1	sema3d	+	1945	1959	5.59E-05	0.722	GGCGCGCGGCCGCTA
SMAD5	MA1557.1	pax2a-201	-	1952	1961	0.00013	1	CGTCTCGCCT
Mad	MA0535.1	pax2a-201	-	1802	1816	0.000206	1	GACACGAGGCTGGTC
GLI2	MA0734.1	pax2a-201	+	1962	1973	0.000323	1	GCGACCTCAGTC
SMAD5	MA1557.1	pax2a-201	-	1194	1203	0.000395	1	CGGCCAGCCA
SMAD5	MA1557.1	pax2a-201	+	1952	1961	0.000476	1	AGGCGAGACG
Mad	MA0535.1	pax2a-202	-	2428	2442	0.000206	1	GACACGAGGCTGGTC
SMAD5	MA1557.1	pax2a-202	-	1820	1829	0.000395	1	CGGCCAGCCA
SMAD5	MA1557.1	pax2a-203	+	882	891	0.000368	1	TGTGTAGCCA
SMAD5	MA1557.1	pax2a-203	-	882	891	0.000421	1	TGGCTACACA
GLI2	MA0734.1	pax2a-203	-	1985	1996	0.000457	1	AGGACCCCACGG
GLI2	MA0734.1	pax2a-205	-	1923	1934	5.45E-05	1	GCTACCACACAC
SMAD5	MA1557.1	pax2a-205	-	262	271	0.00013	1	CGTCTCGCCT
Mad	MA0535.1	pax2a-205	-	112	126	0.000206	1	GACACGAGGCTGGTC
GLI2	MA0734.1	pax2a-205	+	272	283	0.000323	1	GCGACCTCAGTC
GLI2	MA0734.1	pax2a-205	-	1548	1559	0.000349	1	GTTACCACAATA
SMAD5	MA1557.1	pax2a-205	+	262	271	0.000476	1	AGGCGAGACG
SMAD5	MA1557.1	bmp4	+	109	118	0.000271	1	CGTCCAGCCC
Mad	MA0535.1	bmp4	+	784	798	0.000401	1	CTCTCGAGGGAAGTG
Mad	MA0535.1	bmp4	-	2036	2050	0.000475	1	CACTCGAGAACAGCA

MOTIF	WIDTH	BEST POSSIBLE MATCH
GLI2	12	GCGACCACACTG
SMAD5	10	TGTCTAGACA
Mad	15	CAGGCGCCGCCGCCG

Random model letter frequencies (--nrdb--):
A 0.275 C 0.225 G 0.225 T 0.275

FIMO 5.3.0

- The p-value of a motif occurrence is defined as the probability of a random sequence of the same length as the motif matching that position of the sequence.

- The score for the match of a position in a sequence to a motif is computed by summing the appropriate entries from each column of the position-dependent.

- The q-value of a motif occurrence is defined as the false discovery rate if the occurrence is accepted as significant.

Supplementary Methods

In situ hybridization probes

Gene	fwd primer	rev primer
ntn1a	GGATCCGTGTTACGACGAGAAC	CTCGCACACTAGGTTTCCATCC
pax2a	GACATGATCTGCACCTGACCAG	CGTTTTCTCTTTTCGCCGTTGG
fsta	(Heermann et al., 2015)	
ephb2b	CCGACGAGAGTTTCTCACAG	TGATGCTGTCAATGGTACGG
sema3ab	TAATCAGACGCACCTGTATG	ATGACATCATACTGGCCATC
sema3d	TCCCTAGCAGAGATGAGAAA	AAACCTCGATCGGTTATCAG
rgmd	TCAACGCAGATGAGCAGC	CGTATGCCCAAACCTCCAC
lrig1	CGTGTACGCTTGATACGACC	GCTAAGGCCTCTGAATGCTC
shha	ATGCGGCTTTTGACGAGAGTGC	GCTTGAGTTTACTGACATCCC
shhb	CAGTGGCTGCTAAATCAGG	ATACGCTTACTTTGCACTATTTC
slit1a	AACTTCATCCAGGCGATTCC	GATTCTGCAGGTCCTGGAAG
slit2	TCCTGCATTGAAGATGGAGC	AAGGCCTTTGGGAAGTTCAG

sgRNA sequences

sgRNA name	Sequence
ephb2b T1	TTTGGCCCCGTCTCCCGTAA
ephb2b T2	AACAGCCGTACAACCAACGA
ephb2b T3	CGTAGATACCTCCGGGTTTG
ephb2b T4	ACTCCCTTACAGCTTCGTTG
lrig1 T1	GAACGATCTTATTACTGCTC
lrig1 T2	CTCGTAGACTGTTATAGTCC
lrig1 T3	CCAGATTGGCTCCTCACTCG
lrig1 T4	TTCAGCCAGGTGATGCGAGG
rgmd T1	GCACTCTCAACCTGGCTAAG
rgmd T2	ACCCTCCGGTGGCTTGCAAG
rgmd T3	CAGCGTTCCAGGACGGCACG
rgmd T4	CATGGGGGGCTGGTACGGAG
sema3d T1	CACAGGCGTAAACATGGGTC
sema3d T2	TAAACGTGCTGTCCTTGCCC
sema3d T3	CTCGTATCAATACTGAGTAC
sema3d T4	GGTGCCGTCCCAAGCGCAGT

Supplementary Table S2: Sequences of PCR primers used for ISH probe generation. Sequences of sgRNAs used for F0 CRISPR experiments.

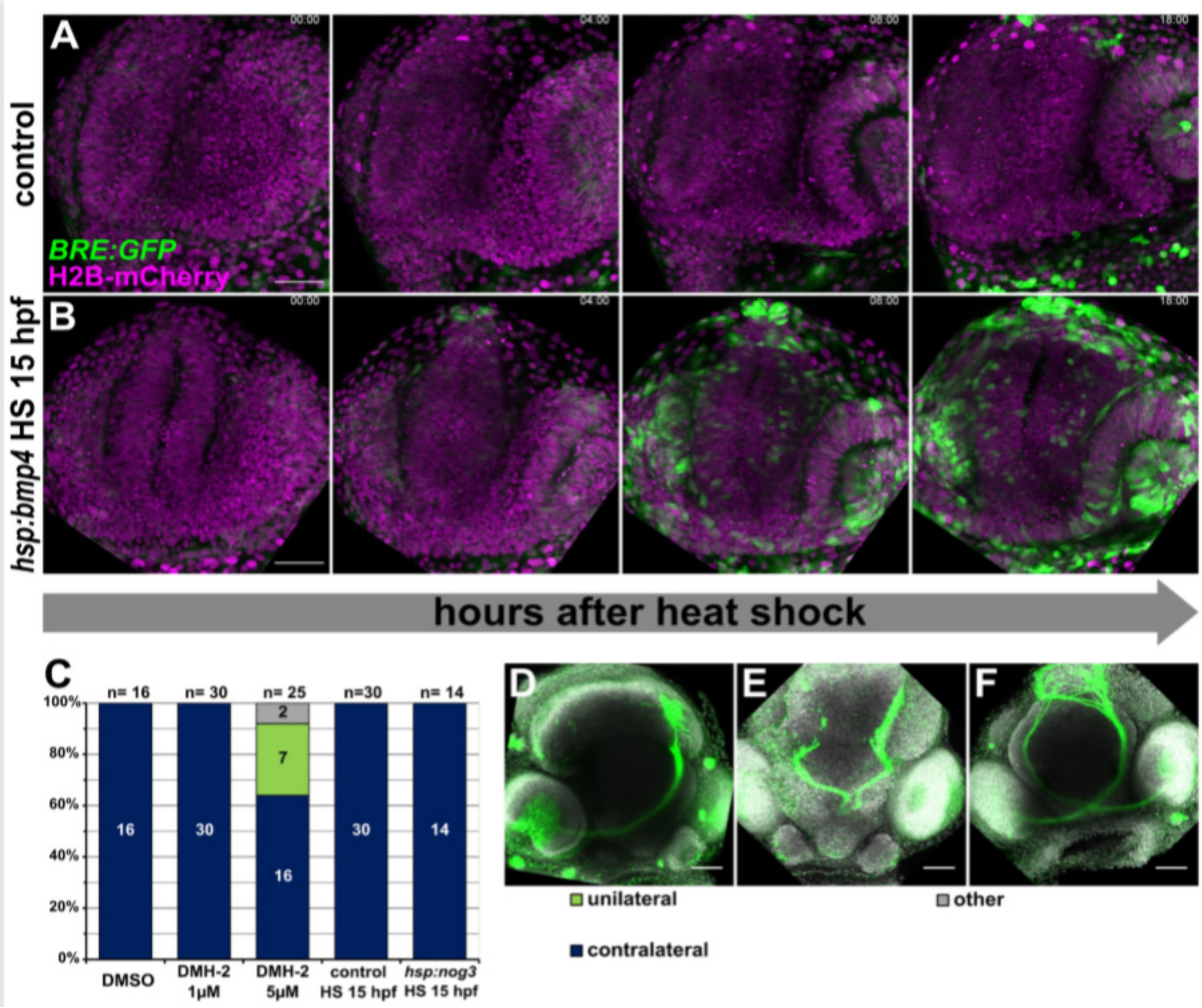

Supplementary Figure S1: BMP signaling reporter is activated by the hsp70l:bmp4 transgene. Inhibition of BMP signaling does not result in ipsilateral RGC projections. (**A**,**B**) Time lapse imaging of (**B**) tg(hsp70l:bmp4, BRE:eGFP, eef1a1:H2B-mCherry) embryos and (**A**) controls after heat shock at 15 hpf. All images are maximum intensity projections, scale bar 50 µm. (**C**) Chart showing the distribution of RGC projection phenotypes in embryos treated with DMH-2 at 15 hpf or tg(hsp70l:nog3) embryos after heat shock at 15 hpf. (**D**–**F**) Phenotypes seen in embryos treated with DMH-2 at 15 hpf. Immunohistochemistry against GFP in tg(pou4f3:mGFP), DAPI counterstaining, 4 dpf. (**D**) Projections absent from one side, (**E**) irregular (contralateral) RGC trajectory and target, (**F**) irregular innervation of optic tecta.

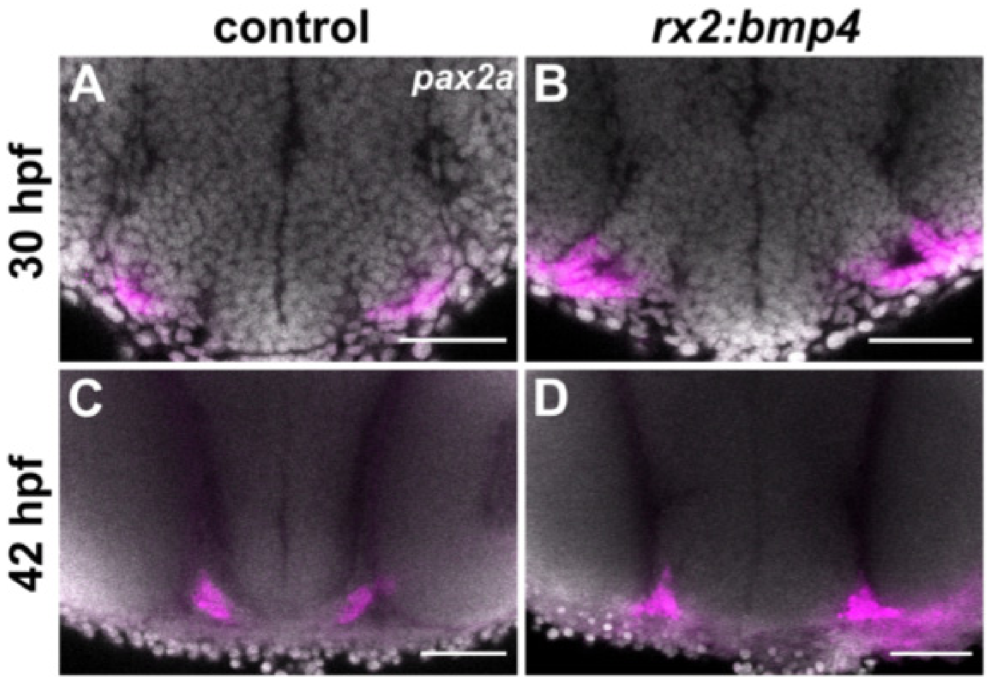

Supplementary Figure S2: pax2a expression in tg(rx2:bmp4). (**A**–**D**) In situ hybridization of pax2a at (**A,B**) 30 hpf and (**C**,**D**) 42 hpf in tg(rx2:bmp4) embryos and controls. All images are maximum intensity projections, DAPI counterstaining, transverse view, scale bars 50 μm.

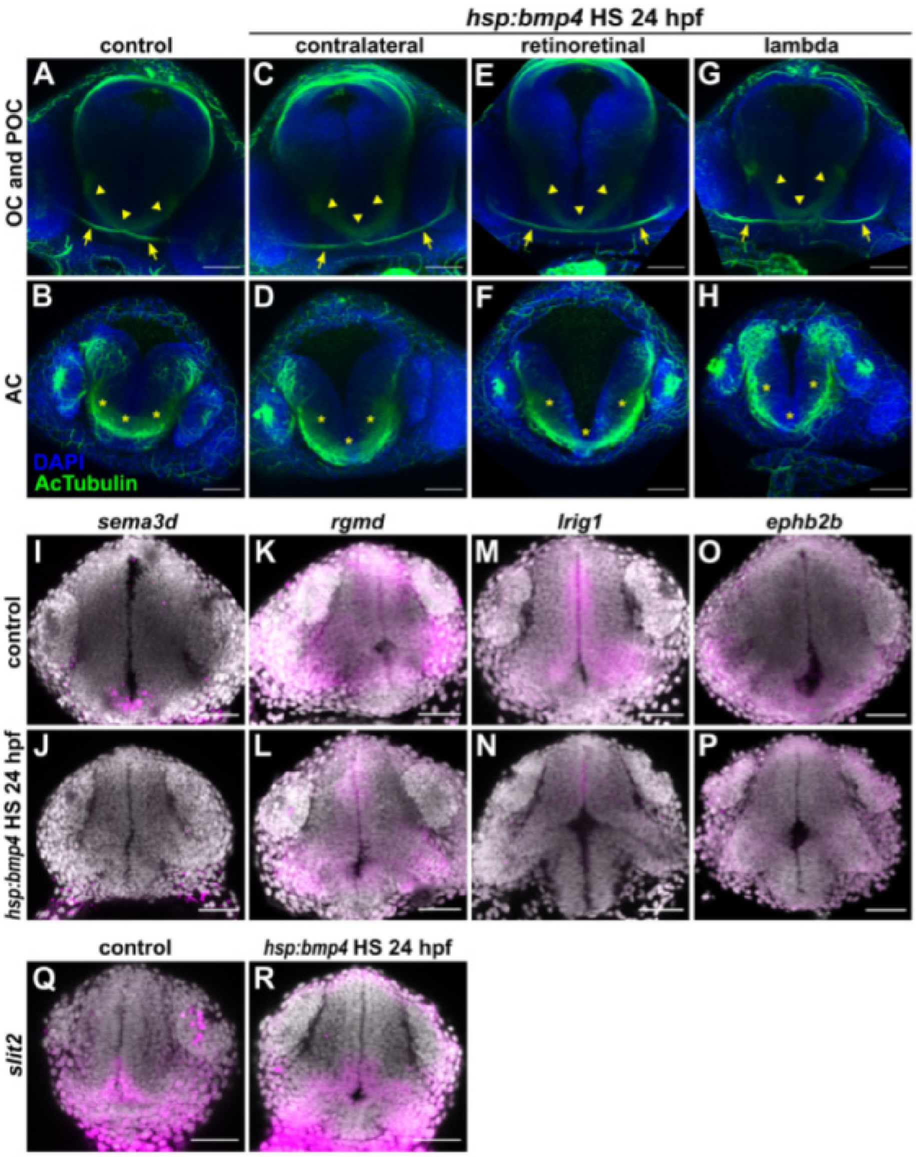

Supplementary Figure S3: Effect of late induction of bmp4 at 24 hpf on forebrain commissures and expression of axon guidance factors. (**A**–**H**) Immunohistochemistry against Ac-αTub in (**C**–**H**) tg(hsp70l:bmp4) embryos and (**A**,**B**) controls at 45 hpf. (**C**,**D**) contralateral phenotype, (**E**,**F**) retinoretinal phenotype, (**G**,**H**) lambda phenotype. (**A**,**C**,**E**,**G**) Post-optic commissure (arrowheads) and optic nerves (arrows); (**B**,**D**,**F**,**H**) anterior commissure (asterisks). (**I**–**R**) In situ hybridization at 30 hpf in (**J**,**L**,**N**,**P**) tg(hsp70l:bmp4) embryos and (**I**,**K**,**M**,**O**) controls after heat shock at 24 hpf. (**I**,**J**) sema3d, (**K**,**L**) rgmd, (**M**,**N**) lrig1, (**O**,**P**) ephb2b, (**Q**,**R**) slit2. All images are maximum intensity projections, DAPI counterstaining, transverse view, scale bars 50μm.
